# Self-Organized Service Negotiation for Collaborative Decision Making

**DOI:** 10.1155/2014/814065

**Published:** 2014-08-27

**Authors:** Bo Zhang, Zhenhua Huang, Ziming Zheng

**Affiliations:** ^1^Department of Computer Science, Shanghai Normal University, Shanghai 201418, China; ^2^Department of Computer Science, Tongji University, Shanghai 200092, China; ^3^Department of Computer Science, Illinois Institute of Technology, Chicago, IL 60616, USA

## Abstract

This paper proposes a self-organized service negotiation method for CDM in intelligent and automatic manners. It mainly includes three phases: semantic-based capacity evaluation for the CDM sponsor, trust computation of the CDM organization, and negotiation selection of the decision-making service provider (DMSP). In the first phase, the CDM sponsor produces the formal semantic description of the complex decision task for DMSP and computes the capacity evaluation values according to participator instructions from different DMSPs. In the second phase, a novel trust computation approach is presented to compute the subjective belief value, the objective reputation value, and the recommended trust value. And in the third phase, based on the capacity evaluation and trust computation, a negotiation mechanism is given to efficiently implement the service selection. The simulation experiment results show that our self-organized service negotiation method is feasible and effective for CDM.

## 1. Introduction

With the increasing complexity of decision-making problems from substantive users, web service-based collaborative decision-making (CDM) technology becomes a feasible solution [1–4]. CDM consists of heterogeneous and geographically distributed organizations with different capacity. It can efficiently combine the most suitable skills from these organizations to achieve a consolidated solution and share the plentiful decision-making resources in open network environments in a cooperated manner. Hence, it can evidently overcome the limitation of the single decision maker [5, 6]. To the best of our knowledge, existing researches of CDM mainly focus on the meeting mechanism, the negotiation protocol, the optimization of decision results, and the management decision-making process [6–10]. However, in a web service-based environment, as an initial action of the collaborative organization, it is important to identify the competent participants of web services for CDM, which has been ignored in existing studies [11, 12].

Web service selection is a challenge problem for CDM. Because the principles of service selection and model selection are similar, traditional efforts are simply devoted to the precious selection from various models [5, 13]. However, in an open and loose-coupling environment, many decision-making service providers (DMSPs) are not free, and the decision-making sponsor generally has insufficient information about all DMSPs. As a result, the sponsor has to accept DMSPs' various payment conditions without any opportunity to experience the services in advance. On the other hand, the sponsor may abandon some high quality DMSPs since it lacks sufficient knowledge to certify these DMSPs' abilities. Such asymmetry position would result in improper and inefficient CDM. To overcome these problems, decision participants request an efficient mechanism to identify qualified partners without the full knowledge about them.

Based on the above facts, in this paper, we propose a new approach for capacity and trust evaluation of DMSPs' services. This proposed method can especially adopt more factors to improve the accuracy and precision of the evaluation. Further, we present an efficient negotiation method for the sponsor to organize CDM and select DMSPs' services automatically. We show in the experiments that our self-organized service negotiation method is feasible and effective. To sum up, our key contributions are as follows.

1. We give the formal semantic description of the complex decision task for DMSP and compute the capacity evaluation values according to participator instructions from different DMSPs, including three aspects: goal evaluation, time forecasting, and costs estimation.
2. We present an efficient approach to compute the trustiness of DMSPs and their services from both subjective and objective facets, called belief and reputation ranking in this paper, respectively. We further present a recommendation-based trust computation method for the CDM sponsor to identify strange DMSPs under recommendation from the sponsor's familiar service providers.
3. We propose a novel strategy of service selection to find out the optimal CDM participants. This paper employs an organization mechanism for the sponsor and DMSPs, which is based on a bidding rule and enables the sponsor to negotiate with DMSPs to select the most competent CDM participants.
4. We develop a prototype system for the collaborative decision-making environment and design four types of examinations to verify the feasibility and effectiveness of our proposed method. The experimental evaluation shows that our proposed method is both feasible and effective.

## 2. Related Works

### 2.1. Collaborative Decision Making

Collaborative decision making has been widely used in many application domains, such as airport management [1, 2], enterprise cooperation [3], and stakeholder research [4]. In practice, the CDM framework is proposed in three ways, that is, Internet based CDM [6, 14], multiagent based CDM [15], and web service-based CDM [16, 17]. Internet based CDM is a traditional way to organize the decision making. The main challenge of Internet based CDM is how to transfer isometric data and information across wide networks. Multiagent is a feasible and optimized solution for CDM. Agent has abilities of negotiation, decision making, and knowledge interaction, which can partially implement intelligent and automatic CDM. However, because agents lack the mechanism of self-description in a machine readable format, it is difficult for agent oriented CDM to understand the characteristics of CDM, such as CDM requirements, capacities, and creditable degrees of candidate partners. Such situation brings about difficulties for identifying qualified decision-making partners. In recent studies, web service becomes a popular solution. Web service is a software program designed to support interoperable machine-to-machine interaction over a network [18]. However, most existing collaborative decision-making methods focus on selecting services directly by a system assigned model and lack interactive methods to enable system to organize collaboration in a negotiation way. That is, CDM can be achieved autonomously through negotiation scheme to obtain better collaboration performance. In this work, our contribution is to propose a CDM organizing method which allows services to negotiate automatically in service oriented architecture.

### 2.2. Model Selection of Decision Making

As the core problem of CDM, DMSP selection is constantly treated as decision model selection in traditional DSS (decision support system). Artificial intelligent (AI) techniques are widely used for model selection, such as CBR (case based reasoning) [19], RBR (rule based reasoning) [20], ANN (artificial neural network) [21], and GA (genetic algorithm) [22]. Statistical methods, such as Bayesian information criteria, are also frequently adopted for decision model selection [23]. Mou et al. proposed a QoS based service selection in CDM [17], where QoS is measured as the capacity of web service. While Mou's model mainly focuses on service capacity forecasting, our trust computation strategy provides a comprehensive solution for efficient service selection.

However, these existing methods are not designed for open and distributed network. In such loose-coupling environment, different providers are allowed to deploy various services. Then, there would be big performance differences among services. That means performance evaluation of decision service is indispensable for service selection. Meanwhile, the reliability measurement is also an essential part of service selection since the risks from malicious services cannot be neglected in the open network environment. In our view, decision-making services are deployed in distributed and risk-existing environment. It is essential for CDM to select those services which have the best competitive capacity and the most trustworthy qualifications. Traditional decision model selection methods paid less attention to the above point, which is the main motivation in this paper.

### 2.3. Trust Computation Research

Trust, as an inherent characteristic of human beings, demonstrates the emotional and logic confidence relationships between individuals [12]. It is derived from the judging of authenticity by the evaluation of various facts that can lead to confidence or distrust. Because trust is a natural disposition of the human brain and also reflects the reliability of individuals, we can describe trust from subjective and objective perspectives. In trust computation, belief and reputation are usually two core concepts for creditable description. Belief is a subjective conception that demonstrates a creditable relationship between two or more individuals. On the other hand, reputation represents the overall common schema from all the qualified members.

A substantial amount of research has been conducted on belief and reputation in the past decades [12, 24–27]. The result has been the proposal of several methods, such as summation/average/iteration of past trust ratings [25, 26] and Bayesian systems [24, 27], to optimize one or more aspects of trust computation performance. On the basis of trust computation, the architecture of reputation systems is categorised into two main types: centralized [12] and distributed [11]. Centralized systems utilize a central authority to collect all ratings and publish reputation scores for every participant, whereas in distributed reputation systems each member acquires belief about each experience with others and submits the reputation on request when it is requested by other members. The majority of WSN nodes are deployed without centralized monitoring. Therefore, assigning a trust center is not a feasible scheme and so distributed trust management is essential for WSNs.

The weighted average of ratings method is a typical trust computation scheme that is extensively utilized [28]. In this method, all trust ratings with respect to the target object are aggregated and the weighted average of the aggregation is calculated as the new trust value for the target object. Technically, the average method of trust is easy to realize if witness information and ratings are available. However, trust ratings aggregation from a long judgment path is not considered as weakening the trust.

The Bellman-Ford algorithm computes trust based on direct witness interaction trust judgments [19]. It generates a trust graph on the basis of the trust link between two peers who have direct interaction. Each peer can submit or renew their trust judgments of others based on new direct interactions. Further, the trust between peers is constantly updated by compounding old and new trust judgments. In addition, the algorithm admits the most trustable path for trust computation; it deems a long path untrustworthy. However, it has no mechanism to prevent loops in the trust path.

Qureshi et al. [11] proposed a robust distributed reputation and trust management scheme, called M-Trust, for peers in mobile networks. The proposed scheme builds reputation based on peer interactions and integrates five characteristics, reliability, accuracy, adaptability, robustness, and light weight, in acquiring and aggregating trust ratings from peers.

Reputation can be considered a collective measure of trustworthiness (in the sense of reliability) based on the referrals or ratings from members within a community [12]. Therefore, it is aggregated from the joint decisions of various members. Several reputation computation methods are currently extensively used. They include the sum and average of ratings [29] and the Bayesian method, which is based on previous reputation knowledge [30]. Chen et al. [31] proposed a local and global average method that integrates personal opinions and public attitude with the reputation of a target. Their proposal exemplifies a type of method that obtains an average reputation from a combination of individual experiences and second-hand referrals.

In our previous research, we proposed a trust computation based model selection for decision support system, which considers the trust from subjective and objective views [32]. But the proposed method only can help system to recognize the decision-making model from the creditable aspects and not the capacities of decision models. We proposed a novel service selection method for CDM in CPS environment [33]. This service selection method is based on both capacity and trust criteria. However, the selection mechanism in CPS was based on both cyber and physical sides and we also realized that the capacity evaluation and trust computation should be revised to fit the web service environment. Furthermore, the selection mechanism was not supported by analysis from examination. In this paper, we improve the computation methods and also give further examinations to testify the effect and feasibility of our selection mechanism in this paper.

## 3. Selection Model of DMSP

**Figure 1:**
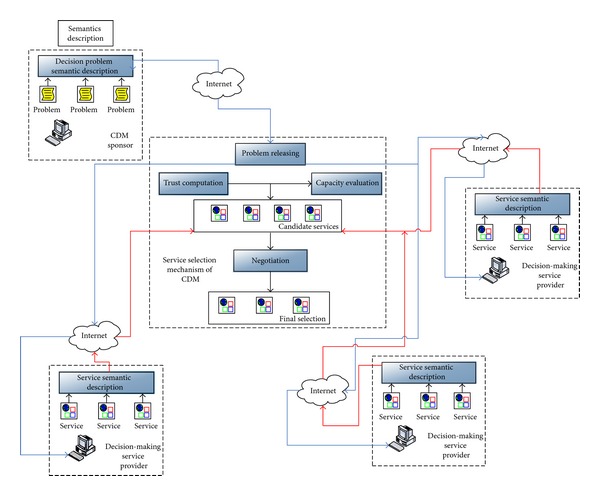
Our DMSP selection mechanism for CDM.

Our DMSP selection mechanism can be shown in Figure 1.

In Figure 1, blue lines denote the releasing of the decision task semantics, and red lines denote the services from DMSPs in the selection process. It is not difficult to see in Figure 1 that there exist three phases: (i) the semantic-based capacity evaluation for the CDM sponsor, (ii) the trust computation of CDM organization, and (iii) the negotiation selection of DMSP. In the first phase, the formal semantic of complex decision task is described by ontology in the CDM sponsor. Each DMSP analyzes the task semantic and generates the participator instruction according to its capacity automatically. And then, DMSP sends the participator instruction to the sponsor. In the second phase, the trust computation is launched after the sponsor receives all the participator instructions from DMSPs. Particularly, the trust computation is comprised of three steps: the belief computation, the reputation ranking, and the recommendation-based trust computation. In the three phases, the CDM sponsor will negotiate with DMSPs and identify the most competent DMSP participants through the trust and capacity criteria.

## 4. Semantic-Based Capacity Evaluation of CDM

### 4.1. Semantic Description

To evaluate the quality of a candidate service, the decision-making sponsor should match the service's capacity with the requirements of its decision tasks.

**Definition 1 .**

The requirement semantic of decision task is a 6-tuple as *ℵ* = (*ℵ* ^*C*^, *ℵ* ^SR^, *ℵ* ^precon^, *ℵ* ^*goal*⁡^, *ℵ* ^time^, and *ℵ* ^cost^). Here *ℵ* ^*C*^, *ℵ* ^SR^, *ℵ* ^precon^, *ℵ* ^*goal*⁡^, *ℵ* ^time^, and *ℵ* ^cost^ represent the task class name, the structure relationships of task, the preconditions, the goals, the time requirements from sponsor, and the decision making cost price, respectively.

**Definition 2 .**

Participator instruction semantic of decision-making service is defined as a 5-tuple as *I* = (*I* ^id^, *I* ^source^, *I* ^class^, *I* ^*goal*⁡^, *I* ^time^, and *I* ^cost^) according to its capacity. The parameter *I* ^id^  denotes the exclusive identification of service. *I* ^source^ indicates the source of service in DMSP. *I* ^class^ is the class of decision task which the service is able to use. *I* ^*goal*⁡^ is a set of anticipated goals which can be achieved by the service. *I* ^time^ represents the time that the service would spend on decision making. *I* ^cost^ describes the costs that the sponsor should pay for the decision-making service.

### 4.2. Capacity Evaluation

The capacity evaluation comprises three aspects: the goal evaluation, the time forecasting, and the costs estimation.

#### 4.2.1. Goal Evaluation

The goal evaluation aims to identify the goals in *ℵ* ^*goal*⁡^ that is achieved by a service according to its *I* ^*goal*⁡^. We define an equalization mapping function between two semantics as follows.

**Definition 3 .**

Let *x* and *y* be the elements in *ℵ* and *I*, respectively. The equalization mapping function *N*(*x*) → *y* is a transfer relationship between *x* and *y*, which represents the equality of two elements on the semantic level.

Let *ℵ* ^*goal*⁡^ = {*ℵ*_1_^*goal*⁡^, *ℵ*_2_^*goal*⁡^,…, *ℵ*_*n*_^*goal*⁡^}. For each subgoal *ℵ*_*i*_^*goal*⁡^, it has a weight *w*_*i*_ with the constraint ∑_*i*=1_^*n*^ *w*_*i*_ = 1. Then the value of goal evaluation can be calculated below:

(1) $$\begin{array}{c} \text{scor}{\text{e}}_{\mathrm{goal}}\left(I^{\text{id}}\right)=\sum w_{N\left(I^{\mathrm{goal}}\right)\to {ℵ}^{\mathrm{goal}}}. \end{array}$$

Since a large number of *I* ^*goal*⁡^ may satisfy the equalization mapping function *N*(*I* ^*goal*⁡^) → *ℵ* ^*goal*⁡^, the decision-making task will be extremely complicated. To solve this problem, we introduce an impact factor calculation approach for the goal evaluation. Let *m* be the number of *I* ^*goal*⁡^ that satisfy *N*(*I* ^*goal*⁡^) → *ℵ* ^*goal*⁡^; then the final value of goal evaluation can be calculated below:

(2) $$\begin{array}{c} \text{valu}{\text{e}}_{\mathrm{goal}}\left(I^{\text{id}}\right)=\text{scor}{\text{e}}_{\mathrm{goal}}\left(I^{\text{id}}\right)\times {\left(\frac{m}{n}\right)}^{\left(1/m\right)}. \end{array}$$

#### 4.2.2. Time Forecasting

The time forecasting aims to decide whether the response time can satisfy the sponsor's requirement. Here the response time is measured as the time interval between the decision beginning and the service.

For the time forecasting, we denote the maximum affording time by $\tilde{T}\cdot {ℵ}_{j}^{\text{time}}$ which indicates the maximum time limit of each subgoal *ℵ*_*i*_^*goal*⁡^ that would be accepted by the decision-making sponsor. Assume that the expected time of each subgoal *ℵ*_*i*_^*goal*⁡^ from the sponsor is $\overset{-}{T}\cdot {ℵ}_{j}^{\text{time}}$. Expected time signifies the longest decision-making spending time that would be afforded by the interval sponsor. Let the set of response times given by the service be *I* ^time^ = {*I*_1_^time^, *I*_2_^time^,…, *I*_*l*_^time^}, and *I*_*j*_^time^ indicates the time cost that would be expended for each subgoal *ℵ*_*j*_^*goal*⁡^ by the service *I*. Then the value of time forecasting can be expressed below:

(3) valuetimeIid=∑j=1lmatchIjtime  +∑j=1lT−·ℵjtime−IjtimeT~·ℵjtime×ℵgoal⁡−1,

where *l* is the cardinality of *I* ^time^ and |*ℵ* ^*goal*⁡^| is the cardinality of *ℵ* ^*goal*⁡^. We also propose a match function match(*I*_*j*_^time^) to calculate the excess time of *I*_*j*_^time^ relative to *ℵ* ^time^ below:

(4) $$\begin{array}{c} \text{match}\left(I_{j}^{\text{time}}\right)=\left\{\begin{array}{cc} 1 & \text{if}\;\;N\left(I_{j}^{\text{time}}\right)⟶\tilde{T}\cdot {ℵ}_{j}^{\text{time}}\\ 0 & \text{else}. \end{array}\right. \end{array}$$

#### 4.2.3. Cost Estimation

The cost estimation aims to test whether the service's cost *I* ^cost^ is overcharge. Like the time forecasting, the less cost service charge for the decision making is, the more value of *I* ^cost^ would be given from the CDM sponsor. We denote the maximum affording cost by $\tilde{C}\cdot {ℵ}_{k}^{\text{cost}}$ which represents the maximum cost limit of each subgoal that would be acceptable. Assume that the expected cost of each subgoal *ℵ*_*i*_^*goal*⁡^ from the sponsor is $\overset{-}{C}\cdot {ℵ}_{k}^{\text{cost}}$.

Let the set of response times given by service be *I* ^cost^ = {*I*_1_^cost^, *I*_2_^cost^,…, *I*_*q*_^cost^}, and *I*_*k*_^cost^ indicates the time cost that would be expended for each subgoal *ℵ*_*k*_^*goal*⁡^ by the service *I*. Then the value of cost evaluation can be calculated below:

(5) valuecostIid=∑k=1qoverIkcost+∑k=1qC−·ℵkcost−Ikcost/C~·ℵkcostℵgoal⁡.

Here, |*ℵ* ^*goal*⁡^| is the cardinality of *ℵ* ^*goal*⁡^ and *q* is the cardinality of *I* ^cost^. We also propose a function over(*I*_*k*_^cost^) to calculate the excess cost of *I*_*k*_^cost^ relative to *ℵ*_*k*_^cost^ as follows:

(6) $$\begin{array}{c} \text{over}\left(I_{k}^{\text{cost}}\right)=\left\{\begin{array}{cc} 1 & \text{if}\;\;N\left(I_{k}^{\text{cost}}\right)⟶\tilde{C}\cdot {ℵ}_{k}^{\text{cost}}\\ 0 & \text{else}. \end{array}\right. \end{array}$$

Based on the goal evaluation, the time forecasting, and the cost evaluation, the capacity evaluation value can be calculated below:

(7) scorecapacityIid=w1×valuegoal⁡Iid+w2×valuetimeIid+w3×valuecostIid.

Here, *w*_1_, *w*_2_, and *w*_3_ are the factors and *w*_1_ + *w*_2_ + *w*_3_ = 1.

## 5. Trust Computation

**Figure 2:**
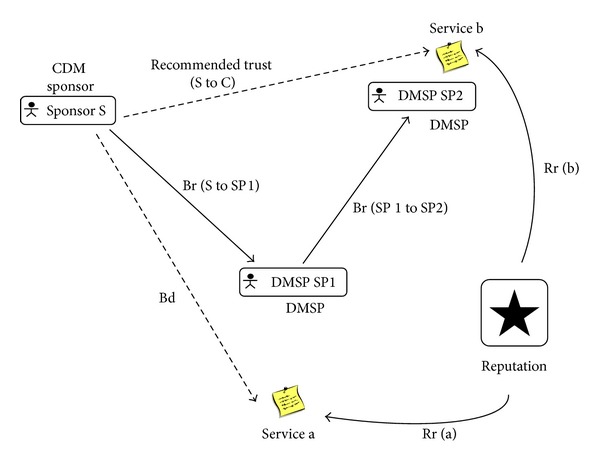
The trust computation of belief, reputation, and recommended trust.

We study the trustable DMSP selection in three aspects: belief, reputation, and recommended trust. Belief is the subjective trust between different DMSPs, which consists of the belief dependence (Bd) and the belief relationship (Br). Belief dependence means the trustable value from the CDM sponsor to candidate services. And belief relationship means the trust relationship value between the CDM sponsor and DMSP.  Figure 2 shows an example of our trust computation framework.

### 5.1. Belief Computation

Let a decision-making service semantic be *I*, which belongs to a DMSP SP. And *I* has made *r* times of decision for the CDM sponsor *R*. Let judge^*R*^(*I*)_*u*_  (judge^*R*^(*I*)_*u*_ ∈ [0,1]) denote the service's score from *R*. Furthermore, we assume that there are *m* times of bad judgments. At the (*r* + 1)th time, Bd from *R* to *I* can be calculated below:

(8) Bdr+1R,I=δ×∑u=1rjudgeRIur×r−mr1/r−m  +1−δ×BrR,SP,r≠0,0.5+BrR,SP2,r=0.

Here, Br(*R*, SP) is the belief relationship value from *R* to SP, which can be calculated by the formula (9). *δ* is a factor whose value is from 0 to 1. And if there is no interaction between *R* and *I*, the value of belief dependence is set to the average of the neutral view (0.5) and Br(*R*, SP).

Like the belief dependence, the belief relationship reflects the whole creditable relationship between the CDM sponsor and DMSP. Assume a DMSP SP has *d* services. Then if all these *d* services have made *t* times of decisions, the belief relationship Br at the (*t* + 1)th time is

(9) Brt+1R,SP=∑v=1dBdR,Ivd×dSP1/t+d,t≠0,0.5,t=0.

In the above formula, |SP| is the total number of services which belong to DMSP. Compared with the traditional average value of reputation computation, our method shows that the more services provided for sponsor are, the higher impact value (*d*/|SP|)^1/(*t*+*d*)^ would be gained for the belief relationship value.

### 5.2. Reputation Ranking

Reputation denotes a public and authoritative trust belief from an adiaphorous community. We build up an independent reputation ranking method to generate impartial reputations for DMSPs.

**Definition 4 .**

Reputation of a DMSP is the summation of evaluation scores from its all past decision making.

Assume a DMSP SP has made *h* times of decision with the evaluation score judge(SP) for the past decision making. Let *g* be the number of sponsors which sent judgments to DMSP in the past. The reputation ranking of SP can be calculated as follows:

(10) RrSP=min⁡∑s=1hjudgeSPsh+0.1×h−gh+g1/h,1.

We use three factors for the reputation ranking: the time limitation, the source identity, and the ranking delay. 

*(i) Time Limitation (T* *L).* We address the first factor named time limitation for reputation ranking. This factor denotes a time period called unit time for DMSP to avoid mass repeated rankings. In this time period DMSP can only receive one appointed number of decision making evaluation from the same CDM sponsor. Let the appointed number of evaluation be TL_*n*_ and let the number of decision makings be TL_*t*_. Then the reputation ranking Rr_TL_(SP)^TL_unit_^ generated by the decision party can be calculated below:

(11) $$\begin{array}{c} {\text{Rr}}_{\text{TL}}{\left(\text{SP}\right)}^{\text{T}{\text{L}}_{\text{unit}}}=\frac{\sum_{j=1}^{\text{T}{\text{L}}_{t}}\sum_{i=1}^{\text{T}{\text{L}}_{n}}\text{judge}{\left(\text{SP}\right)}_{ij}}{i}. \end{array}$$

*(ii) Source Identity (SI).* The reputation ranking should be bound with the evaluation source sponsor's reputation. An evaluation from a source sponsor with a higher reputation generally has more impacts on the DMSP receiver.

Assume there exists a set of sponsors *R* = {*R*_1_, *R*_2_,…}, and each *R*_*i*_ ∈ *R* has sent at least one time of judgment to the DMSP SP. For a CDM sponsor *R*_*i*_ which has the reputation ranking Rr(*R*_*i*_), it sends an evaluation score judge^*R*_*i*_^(SP) to SP. SP will get the evaluation score with the source identity value of *R*_*i*_ as follows:

(12) $$\begin{array}{c} \text{R}{\text{r}}_{\text{SI}}\left(\text{SP}\right)={\text{judge}}^{R_{i}}\left(\text{SP}\right)\times \text{weight}\left(R_{i}\right). \end{array}$$

Let *n* denote the total number of times that *R*_*i*_ has sent judgments to DMSPs, let *m* denote the number of times that *R*_*i*_ has sent judgments to SP in the past, and let max⁡(Rr(*R*)) denote the maximum reputation ranking value of *R*. Then the important degree weight(*R*_*i*_) of *R*_*i*_ can be expressed as follows:

(13) $$\begin{array}{c} \text{weight}\left(R_{i}\right)=\frac{\text{Rr}\left(R_{i}\right)}{\max\left(\text{Rr}\left(R\right)\right)}\times {\left(\frac{m}{n}\right)}^{1/m}. \end{array}$$

*(iii) Ranking Delay (RD).* To determine that a new evaluation score is not an inauthentic evaluation, we use a delay period mechanism. In a delay period, the reputation is just a temporary result (Rr_RD_(SP)), and such reputation can be withdrawn when it is identified as any illegal trick.

Let the time of preserving the evaluation score in a delay period be RD_*t*_ and let the whole length of this delay period be RD_*l*_. Then the temporary ranking can be expressed as

(14) $$\begin{array}{c} \text{R}{\text{r}}_{\text{RD}}\left(\text{SP}\right)=\frac{\text{judge}\left(\text{SP}\right)\cdot {\text{RD}}_{t}}{{\text{RD}}_{l}}. \end{array}$$

From the above three factors, the reputation ranking Rr(SP)^*T*+*t*^ can be expressed as

(15) RrSP1T+t=RrSP1T+∑j=1m ∑i=1njudgeSP1∗weightRrSPij∗li∗t.

Here, *T* and *t* are time point and the delay period, respectively.

### 5.3. Recommendation-Based Trust Relationship Computation

In an open network environment, it is impossible for the decision sponsor to comprehend all the various web services. To understand the strange web services, the sponsor can use the recommendations from their acquaintances. Based on this fact, we introduce a recommended trust to initialize the relationship between the CDM sponsor and the strange DMSP. Recommended trust is built up through an intermediate DMSP which has beliefs with both the CDM sponsor and the strange DMSP.

For the CDM sponsor *R*, the DMSP SP^*E*^, and the set of DMSPs SP^*R*^ = {SP_1_^*R*^, SP_2_^*R*^, SP_3_^*R*^,…, SP_*n*_^*R*^}, if Br(*R*, SP^*E*^) = 0∧Br(*R*, SP_*i*_^*R*^) ≠ 0∧Br(SP_*i*_^*R*^, SP^*E*^) ≠ 0 and ∃*I* ∈ SP^*E*^∧Bd(SP_*i*_^*R*^, *I*) ≠ 0, then the recommended trust (RT(*R*, SP_2_ · *I*)) can be expressed as

(16) RTR,SPE·I=α×∑i=1nBrR,SPiRn+β×∑i=1nBrSPiR,SPEn+γ×∑i=1nBdSPiR,SPE·In,

where *α*, *β*, and *γ* are the parameters which are set by the system.

For the CDM sponsor, the recommended DMSP is an unfamiliar service provider with the full confidence. Hence we propose a confidence conformation factor for the recommended DMSP based on the objective reputation with the impartial nature. We suppose that there exist *d* intermediary DMSPs {SP_1_^*in*^,…, SP_*d*_^*in*^} which recommend the same DMSP SP to the CDM sponsor. Then the confidence conformation factor *ϕ* can be expressed as

(17) $$\begin{array}{c} \phi =\frac{\left(\sum_{i=1}^{d}\left(\text{RT}\left(R,\text{SP}\right)\cdot \text{Rr}\left(\text{S}{\text{P}}_{i}^{in}\right)\right)\right)\cdot \text{Rr}\left(\text{SP}\right)}{\sqrt{\sum_{i=1}^{d}{\left(\text{RT}\left(R,\text{SP}\right)\cdot \text{Rr}\left(\text{S}{\text{P}}_{i}^{in}\right)\right)}^{2}}\cdot \sqrt{\sum_{i=1}^{d}{\left(\text{Rr}\left(\text{SP}\right)\right)}^{2}}}. \end{array}$$

In our consideration, the confidence conformation factor *ϕ* aims to show the similarity between the recommended trust and the recommended DMSP's reputation. Hence, the formula (17) is presented according to the Cosin method which is widely used to calculate the similarity between two vectors.

## 6. Service Negotiation for DMSP

In order to make the best decision, the CDM sponsor always wants to select the most competent services. The capacity and the trust are two critical aspects for candidate services. In this paper, our service selection method is based on the principles of capacity and trust.

First of all, we define a set of message primitives for the negotiation as follows:

- send(): send a message;
- reject(*a*, *b*): inform rejecting the event *a* and send the event *b*;
- send_value(*a*, value): send the value of the event *a*;
- accept(): send a set of acceptable events to the other;
- revise(*a*, *b*): revise the event *a* as *b*;
- query(*a*): query the state of event *a*.

In the following part, we give our service selection method which consists of 12 steps.

1. The CDM sponsor decomposes the complex decision problem according to the structure relationship of the semantic *ℵ* and forms *ħ* subproblem semantics {sub_*ℵ*_1_,…, sub_*ℵ*_*ħ*_} of *ℵ*.
2. For each subproblem semantic sub_*ℵ*_*i*_ (*i* ∈ [1, *ħ*]), consider the following.
3. The CDM sponsor sends sub_*ℵ*_*i*_ to DMSPs which have belief relationships Br(*R*, SP_*k*_) ≥ *ϑ* using the primitive send(sub_*ℵ*_*i*_). And meanwhile DMSPs which receive sub_*ℵ*_*i*_ transmit sub_*ℵ*_*i*_ to the strange ones of the sponsor with well-deserved belief relationships.
4. After DMSPs receive sub_*ℵ*_*i*_, they will send a message Accept(sub_*ℵ*_*i*_) to the CDM sponsor if they want to accept the decision tasks. Otherwise, they will send a message reject(sub_*ℵ*_*i*_) to the CDM sponsor to inform that they want to surrender the opportunity to take part in sub_*ℵ*_*i*_.
5. If a DMSP wants to recommend another DMSP SP to the CDM sponsor, it uses send(SP · *I*) to send a message and recommends the service *I* of SP to the CDM sponsor. After the CDM sponsor receives such recommendation, it will query SP by the primitive query(SP · *I*) and use send(sub_*ℵ*_*i*_) to confirm whether SP will take part in the collaborative decision making. If SP reply “yes,” then the CDM sponsor will inform the decision tasks.
6. All the affirmed services from each different DMSP SP_*k*_ send their service semantics using send(*I*_*j*_) to the CDM sponsor. For each candidate service semantic *I*_*j*_, the CDM sponsor computes the evaluation scores of score_capacity_(*I*_*j*_), Bd^*R*^(*I*_*j*_), and Rr(SP_*k*_). Moreover, the CDM sponsor computes the score of RT^*R*^(*I*_*j*_) for each recommended service.
7. The CDM sponsor selects candidate services *I*_*j*_ for sub_*ℵ*_*i*_ with score_capacity_(*I*_*j*_) ≥ *ζ*. Particularly, if no *I*_*j*_ is selected for sub_*ℵ*_*i*_, the CDM sponsor selects the one which has the maximum value of score_capacity_(*I*_*j*_). The selected services are put in a set Γ.
8. The CDM sponsor sends the message reject(*I*_*j*_) to each DMSP whose services are not in Γ.
9. For each service in* Γ*, the CDM sponsor sends the message revise(*I*_*j*_^id^, plan) to the corresponding DMSP to ask for the detailed revision plan.
10. When the DMSP receives the revision claims, it will determine whether to modify its plan. If the DMSP modifies the plan, it sends the new plan using revise(*R*, plan) to the CDM sponsor. Otherwise, it sends the rejection claim reject(*R*, plan) to the CDM sponsor.
11. The CDM sponsor repeats the negotiation steps (8) and (9) until at least one service in Γ modifies its plan.
12. The CDM sponsor recomputes all score_capacity_(*I*_*j*_) of the services in Γ after the negotiation and selects each service *I*_*j*_ which satisfies the constraint score_capacity_(*I*_*j*_) + Bd(*R*, *I*_*j*_) ≥ *ρ* or score_capacity_(*I*_*j*_) +RT(*R*, SP^*E*^ · *I*_*j*_) ≥ *ρ*. For the services that do not satisfy this constraint, the CDM sponsor rejects them and removes them from Γ.
13. For sub_*ℵ*_*i*_, the CDM sponsor selects the services *I*_*j*_ as the final victor with the maximum reputation values of the DMSP. If the selected service is a recommended one, the CDM sponsor computes its confidence factor *ϕ*. If *ϕ* is acceptable, then the CDM sponsor ascertains that the recommended service is victor. Otherwise, the CDM sponsor selects the second highest value of reputation.

## 7. Simulation Experiments

We develop a prototype system for the experimental analysis in this work. The prototype of CDM is designed for manufacturing management and marketing decision making in medical manufacturing enterprises. The prototype is deployed in 8 computing nodes in this scenario of simulation. There are 62 DMSPs in the distributed nodes for providing decision-making service. The total number of services is set to 461, including decision-making service types of forecasting, planning, controlling, mining, reasoning, and analyzing in manufacturing, finance, marketing, human resource, and so forth. All services are developed manually and included in DMSPs randomly. In addition, we set initial relations among DMSPs for trust and recommendation computing. The network topology of our prototype is generated according to DMSPs relations and average out-degree of a DMSP is 5. Reputation values of DMSPs are initially set by following a normal distribution with mean 0.8 and variance 0.1 in our prototype. Meanwhile, trust values between DMSPs, which have direct relations, are set initially by calculating their past collaborations according to the collected data. The detailed information is shown in Table 1.

### 7.1. Performance Evaluation of the Trust Computation

In this examination, we validate a set of tests and evaluations to testify the performance of our proposed trust computation methods, including the belief computation, the reputation ranking, and the recommendation-based trust computation. In the following examinations, the parameter *δ* in (8) is set to 0.8.

#### 7.1.1. Performance Evaluation of the Belief Computation

In this examination, we set three tests to validate the effects of our belief computation.

**Figure 3:**
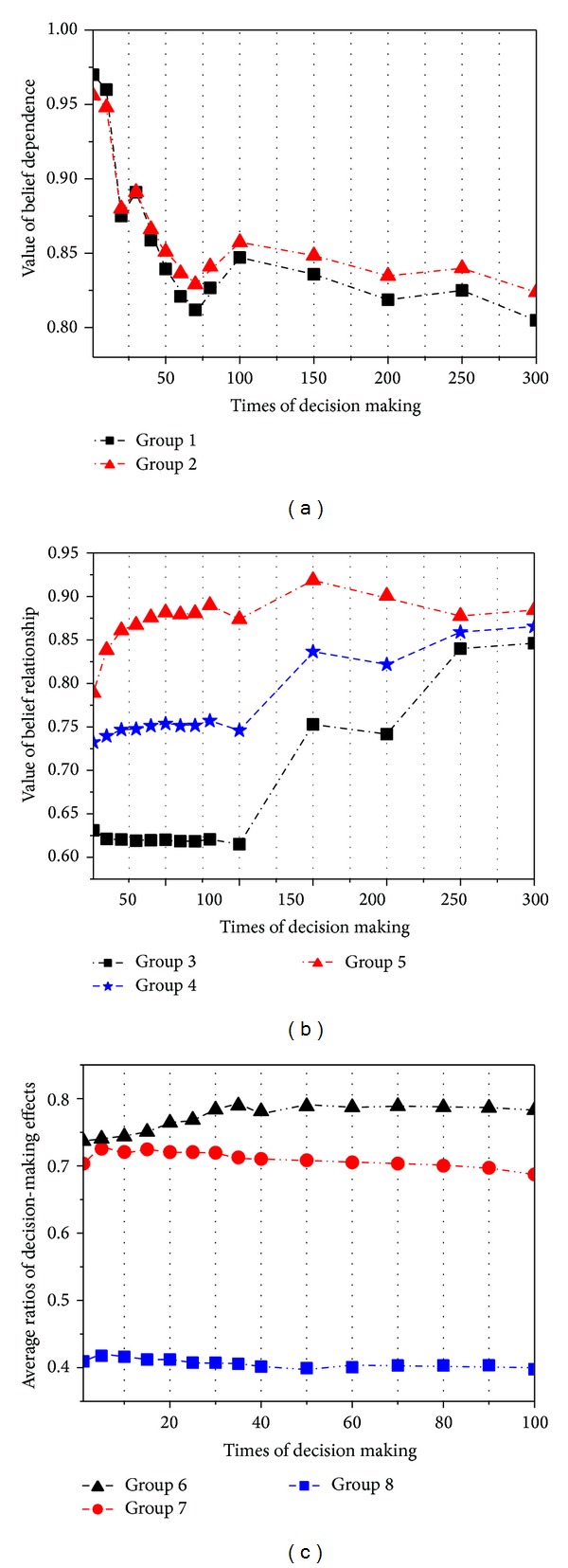
Effects of the belief computation evaluation.

In the first test, we randomly select a CDM sponsor and a DMSP for the belief dependence computation. We appoint a service in the selected DMSP to make decision for the CDM sponsor. We set two groups of computation methods as follows: (1) Group 1 uses the average of past interaction experience to calculate the belief dependence between the CDM sponsor and the service (such method was first proposed in [10] as a trust model EigenRep) and (2) Group 2 uses our computation method in this paper. We repeat the decision making 300 times and record the belief dependence value, which is shown in Figure 3(a).

In this test, we assume that the belief relationship value between the CDM sponsor and the DMSP is a constant value and the chance of bad judgment is below 5%. From Figure 3(a), we can see that the value of belief dependence in Group 1 is lower than that in Group 2. We think that, in Group 1, all interactions are regarded as the same one and have equal judgment efficiencies. However, the computation of belief dependence is influenced by the numbers of decision-making times and the bad judgment provided by the service in Group 2.

In the second test, we randomly select a CDM sponsor and a DMSP for the belief relationship calculation. We appoint different numbers of services in the selected DMSP to make decision for the CDM sponsor. We set three groups of computation methods as follows: (1) Group 3 uses the average of past judgments of all services to calculate the belief dependence between the CDM sponsor and the service, (2) Group 4 uses the average of our belief dependence computation in this paper, and (3) Group 5 uses our belief relationship computation in this paper.

We also repeat the decision making 300 times and record the belief relationship value, which is shown in Figure 3(b). In the first 100 times of decision making, we appoint 30% DMSP's services for the CDM sponsor. And the ratios of services are 60% and 90% in the second 100 and the last 100 times for the CDM sponsor, respectively. From Figure 3(b), we can see that the value of belief relationship in Group 1 is obviously higher than that in the other two groups in the first 30 times of decision making. Such result shows that our method reflects the following situation: the more numbers of services which make decision for the CDM sponsor in a DMSP are, the higher belief relationship value will be got between the CDM sponsor and the DMSP.

In the third test, we assign one CDM sponsor in the prototype system. The CDM sponsor executes 100 times of decision making with different decision tasks. In each task, we provide a certain number of candidate services belonging to different DMSPs which have the ability to solve the task. We compute the average ratio ratio_1 of the decision-making values judge(*I*) from manual operations after each examination:

(18) $$\begin{array}{c} {\text{ratio}}_{1}=\frac{\sum_{g=1}^{100}\text{judge}\left(I_{g}\right)}{g}. \end{array}$$

We study the average ratios in three groups: (1) in Group 6, the CDM sponsor just randomly selects a service from the candidate ones; (2) in Group 7, we adopt the probabilistic model selection mechanism, which can be regarded as a web service mechanism and is widely simulated in the traditional decision model selection [6]; (3) in Group 8, we adopt our method of the belief computation, which integrates the belief dependence (Bd) and the belief relationship (Br) below:

(19) $$\begin{array}{c} \text{select}\left(I\right)=\frac{\text{Bd}\left(I\right)+\text{Br}\left(\text{SP}\right)}{2}. \end{array}$$

Figure 3(c) shows the results of this examination. The average ratios of three groups are 0.406, 0.71, and 0.77. This examination shows that our method outperforms the existing works in all cases. Group 6 adopts a random selection for the decision making, which results in an inaccurate service selection for the appointed decision task. As a result, the decision results in Group 6 are unacceptable. In Group 7, the probabilistic selection achieves a stable average ratio of decision making, which is higher than our method when the number of decision makings is less than 30. The main reason is that our belief computation is based on transaction experiences between the CDM sponsor and the DMSP. As a result, its average ratio is lower than that in Group 7 at the beginning. However, with the increase of decision-making interactions, the CDM sponsor can select the most creditable service with high confidence based on the value judge(*I*). Hence, the probability to select services with better decision-making capacity is high. Eventually the ratio in Group 8 is higher than that in Group 7 when the number of decision makings is larger than 30. This examination shows that our belief computation is feasible and effective.

#### 7.1.2. Performance Evaluation of the Reputation Ranking Computation

In this examination, we implement two tests for the reputation ranking computation.

In the first test, we randomly appoint a DMSP in our prototype to make decision for the CDM sponsor. We set two groups of computation methods as follows: (1) Group 9 uses the average of judgments from the CDM sponsor to compute the reputation ranking, which is widely used in the reputation research, and (2) Group 10 uses our reputation ranking method in this paper.

**Figure 4:**
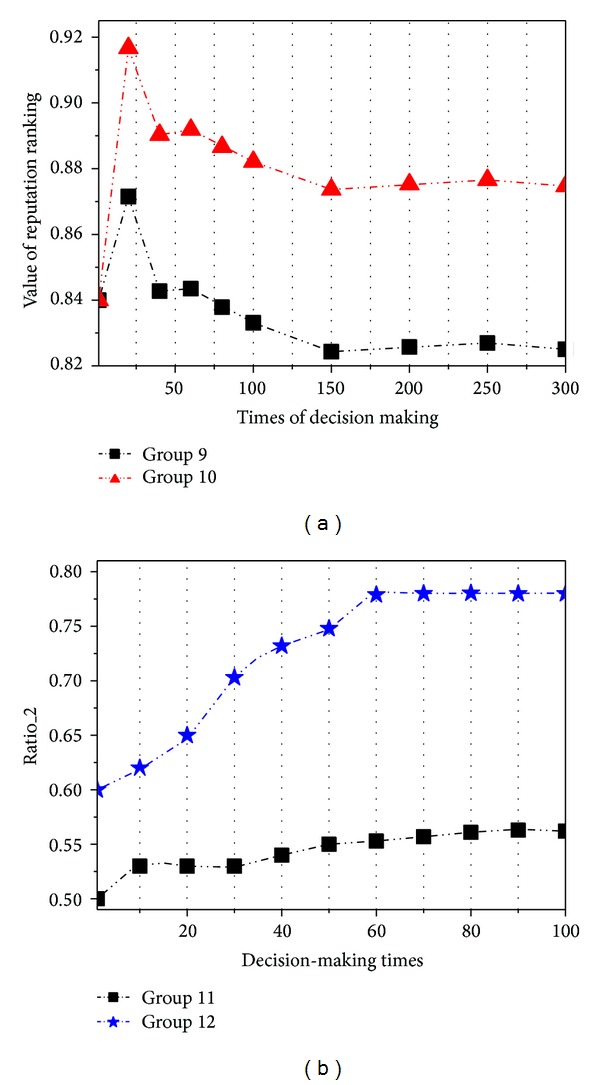
Effects of reputation computation evaluation.

We record the value of reputation in these two groups as shown in Figure 4(a). Figure 4(a) shows that the value of reputation ranking in Group 10 is higher than that in Group 9. This result indicates the following situation: the more number of CDM sponsors which evaluate the DMSP and the more time of decision-making services provided by the DMSP are, the higher reputation will be gained for the DMSP.

In the second test, we define two types of DMSPs: 10 authentic DMSPs and 10 vicious DMSPs. And each DMSP has 10 services. For each authentic DMSP AD, it is a service provider with the real capacities to make decision for the CDM sponsor. However, for each vicious DMSP VD, it is a service provider which sends the vicious and fraudulent evaluation judge(*I*) but does not have any made decisions at all. At the beginning of examination, the reputation ranking values of AD and VD are set to the same scores. We let the CDM sponsor make 100 times of decision tasks which can be resolved by AD. In each time of decision making, the CDM sponsor selects 10 candidate services. The selection criterion integrates the capacity score and reputation rank (Rr) as follows:

(20) $$\begin{array}{c} \text{select}\left(I\right)=\frac{\text{scor}{\text{e}}_{\text{capacity}}\left(I\right)+\text{Rr}\left(\text{SP}\right)}{2}. \end{array}$$

After the decision making, the CDM sponsor provides the evaluations to the selected services. Note that if a service belonging to some vicious DMSP VD is selected for the decision making, VD will send a cheating evaluation score to this service as well.

We set two groups of computation methods for performance evaluation: (1) Group 11 directly calculates the reputation by (10) without involving the time limitation, the source identity, and the ranking delay and (2) Group 12 adopts our proposed method.

We record the accuracy of the service selection of two groups as follows:

(21) $$\begin{array}{c} \text{ratio}\_2=\frac{\sum_{i=1}^{100}\left(\left|\text{AD}\cdot I\right|/5\right)}{i}. \end{array}$$

As shown in Figure 4(b), the accuracy in Group 11 is lower than that in Group 12. Obviously, our method of the reputation ranking can significantly reduce the impact of cheating actions from vicious DMSPs.

#### 7.1.3. Performance Evaluation of the Recommendation-Based Trust Computation

In this subsection, we set three types of examinations to testify the effect of our method for the recommendation-based trust computation, including the parameter evaluation, the malicious recommendation identification, and the recommendation trust precision.

**Figure 5:**
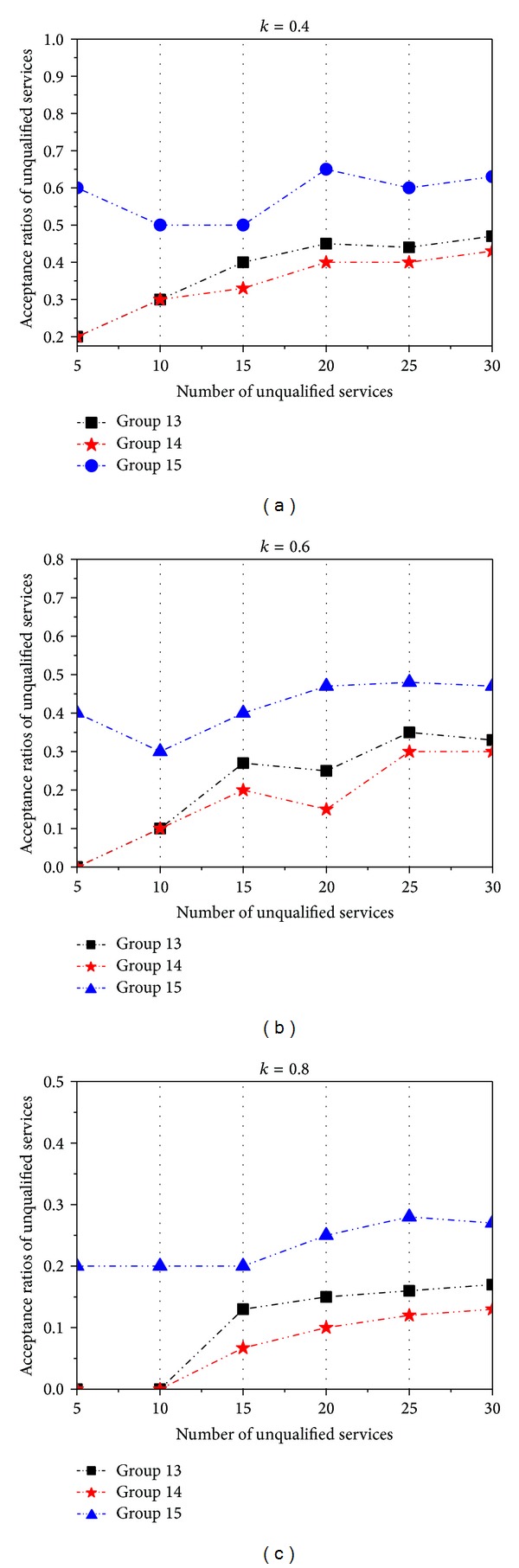
Effects of recommended trust computation parameter evaluation.

In the first examination, we aim to testify the experimental results when the three parameters *α*, *β*, and *γ* in (16) are assigned different values. For all CDM sponsors receiving the recommended services, they must accept the recommendation from intermediates when the recommended trust value satisfies the condition RT(*R*, SP^*E*^ · *I*) ≥ *k*. In this examination, there are 13 DMSPs and 47 services for recommendation. And meanwhile, we assign some unqualified services and assume that there are no malicious intermediates. We implement three groups of computation methods for performance evaluation, Groups 13~15, and set the parameters *α*, *β*, and *γ* to 〈0.2,0.2,0.6〉, 〈0.3,0.3,0.4〉, and 〈0.4,0.4,0.2〉 for these three groups, respectively. We arrange intermediates to recommend unqualified services to CDM sponsors and record different acceptance ratios with the increase of the number of unqualified services. The results are shown in Figure 5.

From Figure 5, we can see that the acceptance ratios of unqualified services decrease with the increase of *k*. And we can further observe that, for the same number of unqualified services, the acceptance ratio in Group 14 is lower than that in the other two groups. We think that these three parameters represent the following three confidences: the CDM sponsor → intermediates, intermediates → the DMSP, and intermediates → DMSP's service quality. And all these confidences should be emphasized in the recommendation-based trust computation. From this point, we can find that the effects of recommendation in Group 15 are better than those in the other two groups.

In the second examination, we aim to test whether our computation method can find out and avoid malicious service providers. The percent of malicious DMSPs varies from 0 to 30%. And for each malicious DMSP, it totally has about 40% unqualified services and its reputation ranking value is lower than 0.3. We set two groups for our examination as follows: (1) Group 16 utilizes the EigenRep method and a malicious recommendation in this group is defined as the one satisfying the condition trust(SP · *I*) ≤ *l* (here trust(SP · *I*) is calculated by the indirect trust computation method in EigenRep) and (2) Group 17 uses our recommendation-based trust computation method. We define that a malicious recommendation is the one satisfying the condition (RT(*R*, SP^*E*^ · *I*) ≤ *k*)∨(*ϕ* ≤ *q*).

**Figure 6:**
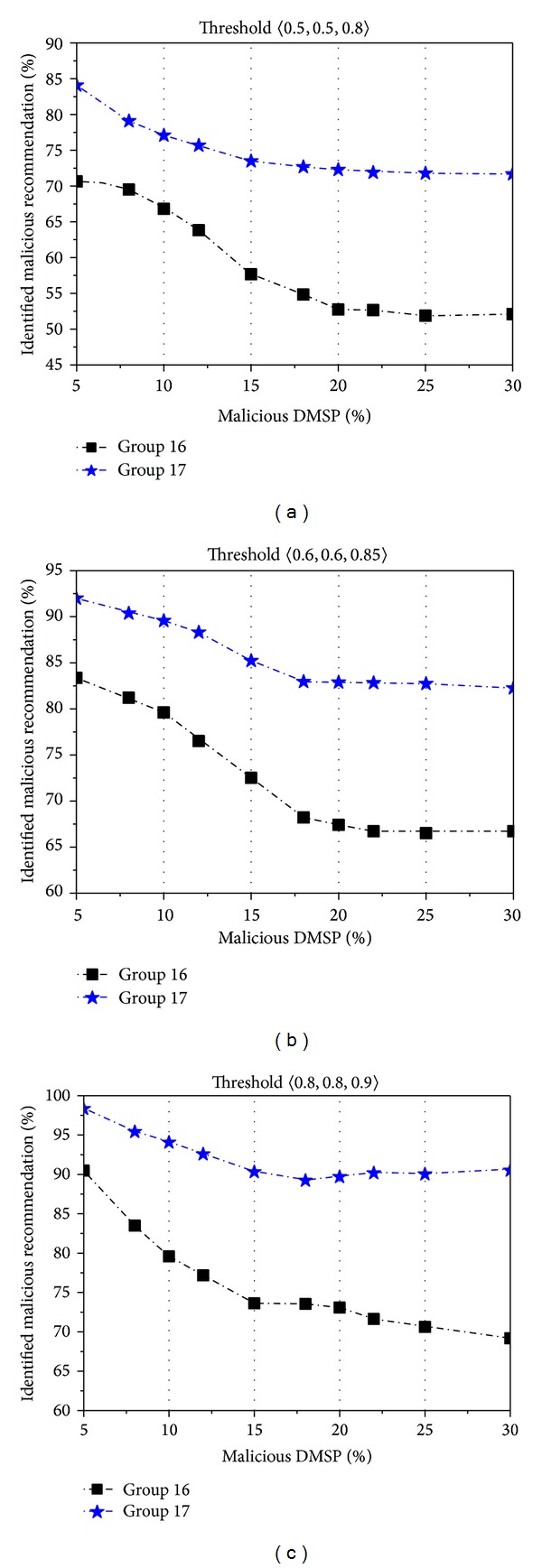
Identification of malicious recommendations.

In each of these two groups, the thresholds 〈*l*, *k*, *q*〉 are, respectively, set to the following three values: 〈0.5,0.5,0.8〉, 〈0.6,0.6,0.85〉, and 〈0.8,0.8,0.9〉. We repeat 50 times of service recommendation under different percents of malicious providers, and the experimental results are shown in Figure 6. From Figure 6, we can observe that the percents of identified malicious recommendations increase with the increase of thresholds in these two groups. This means that our method can efficiently avoid the malicious recommendations.

In the third examination, we focus on testifying the precision of our recommendation-based trust computation. In this examination, we define a precision ratio for the comparison between Group 16 and Group 17:

(22) $$\begin{array}{c} \text{prec}\_f\left(\text{SP}\cdot I\right)=\frac{\left|\text{accepted}\_\text{rec}\right|}{\left|\text{manual}\right|}, \end{array}$$

where |accepted_rec| is the number of services which are accepted by CDM sponsors using our computation method and |manual| is the number of services which can be accepted by CDM sponsors using the manual selection in advance. Therefore, |manual| implies the optimal results in the service recommendation.

In this examination, we randomly select DMSPs to recommend the different services to CDM sponsors and record the precision ratio prec_*f*(SP · *I*). We implement three groups for our examination as follows: (1) Group 18 utilizes the EigenRep method to calculate the indirect trust and determine whether the recommended services can be accepted by CDM sponsors, (2) Group 19 utilizes the kNN (k nearest neighbor) method which is widely used in the collaborative filtering recommendation systems, and (3) Group 20 utilizes our presented method in this paper.

We set the thresholds to trust(SP · *I*) ≥ 0.7 in Group 18 and (RT(*R*, SP^*E*^ · *I*) ≥ 0.7)∧(*ϕ* ≥ 0.9) in Group 20. Furthermore, we carry out three types of tests in each group:

- no malicious DMSPs;
- 20% of the DMSPs are malicious;
- 40% of the DMSPs are malicious.

**Figure 7:**
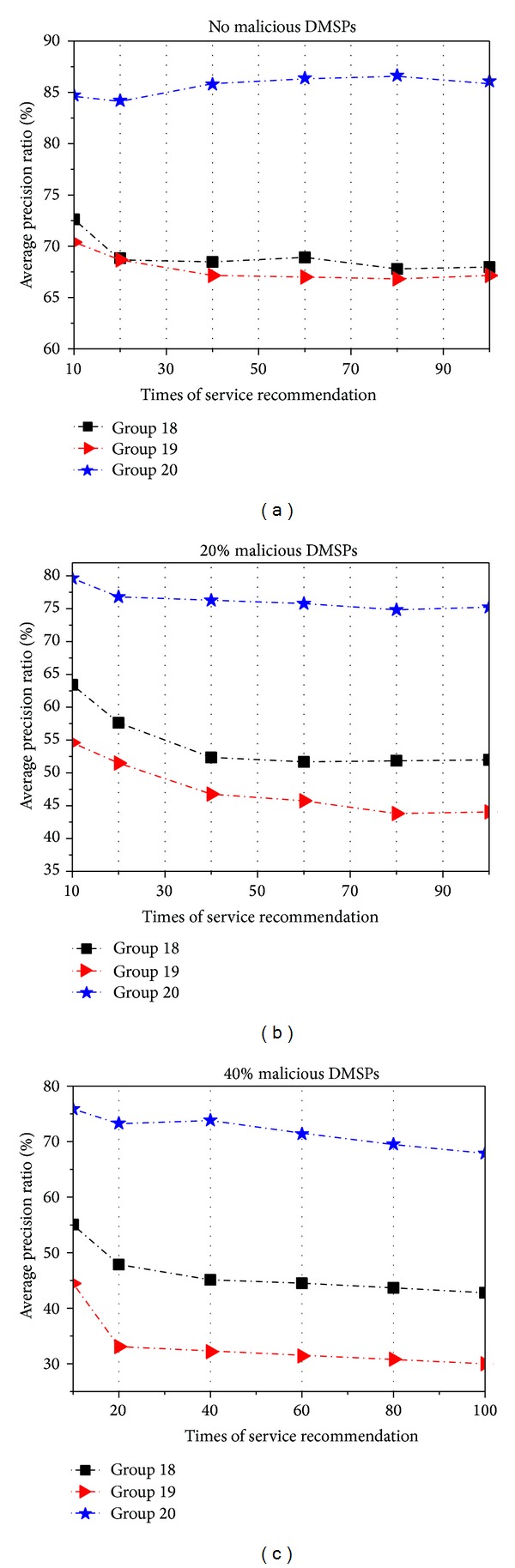
Effects of recommendation-based trust computation for the service selection.

We repeat each test 100 times of recommendation and then record the average precision ratio of service recommendations. The results are shown in Figure 7.

From Figure 7, we can observe that the average precision ratio produced by our method is higher than those produced by the other two methods. We think that the competent service via multiple recommenders will gain the higher recommended trust value using our method. And with the increase of the number of malicious DMSPs, CDM sponsors will receive more unqualified service recommendations in Groups 18 and 19. And it will cause the average precision ratio to decrease.

### 7.2. Performance Evaluation of the Capacity Computation

In this examination, we evaluate the effects of our capacity computation method. We arrange a set of DMSPs to provide their services for the CDM sponsor. The parameters *w*_1_, *w*_2_, and *w*_3_ in (7) are set to the same value 1/3. *δ* in (8) is set to 0.8. And the parameters *α*, *β*, and *γ* in (16) are set to 0.3, 0.3, and 0.4, respectively. Furthermore, the thresholds *ϑ*, *ζ*, and *ρ* in our method are set to 0.4, 0.5, and 1, respectively.

In the examination, there are 13 DMSPs and 47 services for the capacity evaluation. We implement four groups in this examination as follows: (1) Group 21 uses the goal oriented method which selects the services with the most number of goals satisfying the decision requirements, (2) Group 22 uses the time priority method which allows the CDM sponsor to select the services which can maximally satisfy the time requirements of its subgoals, (3) Group 23 uses the lowest price method which allows the CDM sponsor to select the services which have the minimal cost for its subgoals, and (4) Group 24 uses the comprehensive capacity computation method in this paper for the service selection.

We carry out three types of tests in each group:

- no malicious DMSPs;
- 20% of the DMSPs are malicious;
- 40% of the DMSPs are malicious.

**Figure 8:**
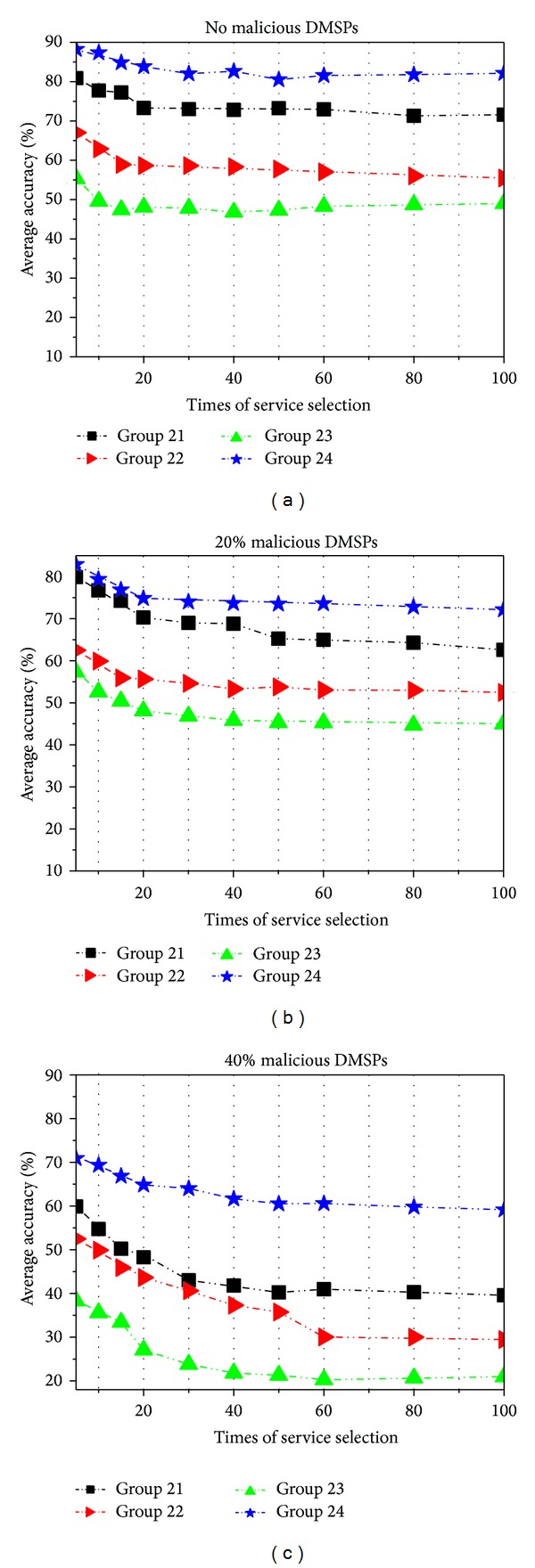
Effects of the capacity evaluation.

Each test is repeated 100 times for the service capacity evaluation. At each time of evaluation, we pick up the optimal services for the requirements of each CDM sponsor in advance. And then we record the average accuracy of service selection using different evaluation methods. The results are shown in Figure 8.

From Figure 8, we can see that the average accuracy of service selection in Group 24 is obviously higher than those in the other three groups. The main reason is that our method is a comprehensive evaluation which includes more selection criteria than other methods.

### 7.3. Performance Evaluation of Our Service Negotiation Method

In this examination, we study the effects of our service selection method which integrates the capacity evaluation, the trust computation, and the negotiation. We use our prototype to execute 100 times of decision making tasks. We implement four groups in this examination as follows: (1) in Group 25, the CDM sponsors select the services with the highest belief dependence value from the candidate services; (2) in Group 26, the CDM sponsors select the services with the highest capacity value from the candidate services; (3) in Group 27, the CDM sponsors select the services using the probabilistic mechanism; and (4) in Group 28, the CDM sponsors select the services using our service selection method. And we record two criteria: the average accuracy of service selection and the average judgment ratio.

**Figure 9:**
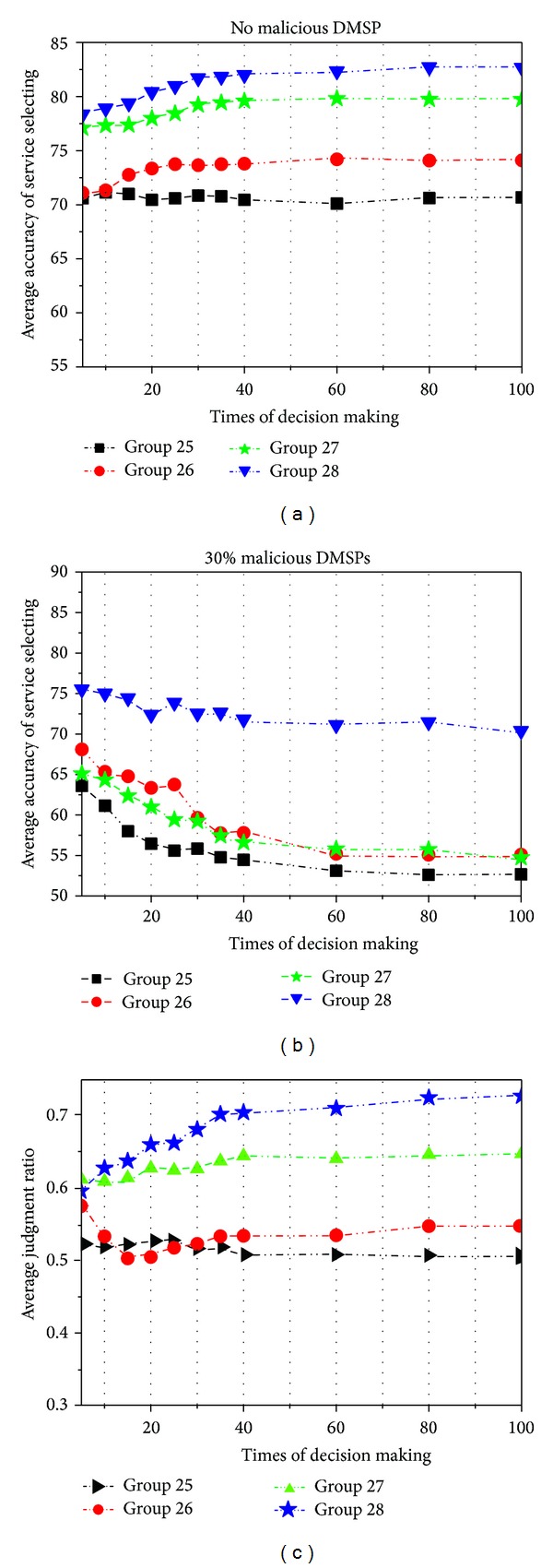
Effects of our service selection method.

In Figures 9(a) and 9(b), we carry out three types of tests to record the average accuracy of service selection in each group:

- no malicious DMSPs;
- 30% of the DMSPs are malicious.

From these tests, we can see that the accuracy under the single capacity or trust criterion is lower than that of our method because our method considers two important aspects: trust and capacity. Moreover, since the mechanism of negotiation and recommendation allows the CDM sponsors to recognize strange DMSPs via recommenders' confidences in our method, the accuracy in Group 28 is higher than that in Group 27.

In Figure 9(c), we can see that our service selection method achieves the best performance in most cases. We notice that the CDM sponsors can only select the services which have high capacity values in Groups 25 and 26. Thus, the CDM sponsors will have a limited scope of service selection and ignore the judgments of trustiness and capacities in Groups 25 and 26. As a result, their average ratios are obviously lower than those of the other two groups. On the other hand, we can see that the average ratio of our method in Group 28 is nearly lower than in Group 27 at the beginning. Nevertheless, with the growth of the number of decision makings, the CDM sponsors and DMSPs will have more opportunities for the collaborative decision making. As the CDM sponsors and DMSPs get more knowledge about each other, the effectiveness of selections is significantly improved. The average ratio in Group 28 finally achieves 0.729, which is higher than the maximum value of 0.649 in Group 27.

### 7.4. Statistical Analysis of the Results

In our simulation, we set the above three experiments for verifying the feasibility and effectiveness of the proposed method in this work. The performances of our proposed trust computation, capacity evaluation, and negotiation based CDM are seen in the results of the above three experiments. Here, we give a statistical analysis of the results for our simulation.

In trust computation experiment, the mean scores of accuracies of belief, reputation ranking, and recommendation-based trust computation for all DMSPs and services in our prototype are 0.79, 0.86, and 0.77, respectively. And we also notice that the variance scores of accuracies decreased with the interactions among services and DMSPs increasing and remained around 0.26, 0.127, and 0.277, respectively, in normal CDM. That is, the accuracies would have less volatility after sufficient interactions.

In capacity evaluation experiment, the mean score of accuracy of service capacity evaluation is around 0.78 for all services deployed in our prototype. We also recorded that the variance score of accuracy of capacity evaluation is around 0.26.

In service negotiation evaluation experiment, the mean score of service selection accuracy is around 0.86 and the variance score of service selection accuracy is around 0.21. In our statistical result, the mean score is low in the beginning phase, while the variance score is relatively high. We consider that the reasons are as follows: (1) most DMSPs have not established effective trust relations toward each other; (2) reputation ranking cannot reflect the authentic trustworthiness since there is not enough judgment in beginning phase; (3) there are few available past performance data for capacity evaluation. All the above reasons lead to inaccurate service negotiation results.

Based on the above statistical analysis, we find that the cold start problem is a significant problem for our proposed method. That means the performance of the proposed method depends on sufficient past data. Therefore, we must focus on how to improve the performance of the proposed method at the beginning phase of service negotiation in CDM because it is difficult to the best performances of capacity evaluation and trust computation while there are few past data for the above computation.

## 8. Conclusion

Web service-based CDM now faces the embarrassment to identify the most competent ones from candidate services. This is due to lack of sufficient prior knowledge for a specified decision making. As a complement of decision-making capacity, the trustable degree of services is of paramount importance because it can judge the authenticity and reliability of strange services with a view of trust. In this paper, we utilize the trust computation for the service selection in the CDM organization. Our method is comprised of three phases. Firstly, the capacity evaluation of services is achieved using the formal semantic description of the decision problem and services. Secondly, we propose the trust computation which involves three aspects: subjective belief trust, objective reputation, and recommended trust. Based on the above two evaluation criteria, we present an automatic negotiation method between the CDM sponsors and DMSPs for service selection. Experimental results show that our negotiation method is feasible and effective.

## Figures and Tables

**Table 1:** The detailed information about our simulation.

Parameter	Value	Type of decision-making service	Value
Number of physical computing nodes	8	Number of forecasting services	74
Number of DMSPs	62	Number of planning services	67
Number of decision-making services	461	Number of mining services	102
DMSPs deployed	Random	Number of controlling services	76
Average out-degree of DMSPs	5	Number of reasoning services	38
Average initial reputation of normal DMSPs	0.8	Number of analyzing services	83
Topology of network	Immutable	Number of workflow services	21
